# Heart failure and depression: A perspective from bibliometric analysis

**DOI:** 10.3389/fpsyt.2023.1086638

**Published:** 2023-03-02

**Authors:** Mei Ieng Lam, Pan Chen, Xiao-Meng Xie, Grace K. I. Lok, Yu-Fei Liu, Tong Leong Si, Gabor S. Ungvari, Chee H. Ng, Yu-Tao Xiang

**Affiliations:** ^1^Unit of Psychiatry, Department of Public Health and Medicinal Administration, & Institute of Translational Medicine, Faculty of Health Sciences, University of Macau, Taipa, Macao SAR, China; ^2^Kiang Wu Nursing College of Macau, Macao, Macao SAR, China; ^3^Centre for Cognitive and Brain Sciences, University of Macau, Macao, Macao SAR, China; ^4^The National Clinical Research Center for Mental Disorders & Beijing Key Laboratory of Mental Disorders, Beijing Anding Hospital & the Advanced Innovation Center for Human Brain Protection, Capital Medical University, Beijing, China; ^5^University of Notre Dame Australia, Fremantle, WA, Australia; ^6^Division of Psychiatry, School of Medicine, University of Western Australia /Graylands Hospital, Perth, WA, Australia; ^7^Department of Psychiatry, The Melbourne Clinic and St Vincent's Hospital, University of Melbourne, Richmond, VIC, Australia

**Keywords:** depression, bibliometrics, hotspots, heart failure, cardiac failure

## Abstract

**Background:**

Depression commonly occurs in heart failure patients, and negatively influences quality of life and disease prognosis. This study explored heart failure and depression-related research from a bibliometric perspective.

**Methods:**

Relevant publications were searched on June 24, 2022. The Bibliometrix package in R was used to conduct quantitative analyses including the trends in publications, and related countries, articles, authors and keywords. VOSviewer software was used to conduct the visualization map on co-word, co-author, and institution co-authorship analyses. CiteSpace software was used to illustrate the top keywords with citation burst.

**Results:**

A total of 8,221 publications in the heart failure and depression-related research field were published between 1983 and 2022. In this field, the United States had the most publications (*N* = 3,013; 36.65%) and highest total citation (*N* = 149, 376), followed by China, Germany, Italy and Japan. Author Moser and Duke University were the most productive author and institution, respectively. Circulation is the most influential journal. Apart from “heart failure” and “depression,” “quality of life,” “mortality” and “myocardial infarction” were the most frequently used keywords in this research area; whereas more recently, “self care” and “anxiety” have been used more frequently.

**Conclusion:**

This bibliometric analysis showed a rapid growth of research related to heart failure and depression from 1989 to 2021, which was mostly led by North America and Europe. Future directions in this research area include issues concerning self-care and anxiety about heart failure. As most of the existing literature were published in English, publications in other languages should be examined in the future.

## 1. Introduction

Heart failure is a complicated clinical syndrome (1) usually caused by cardiovascular and chronic obstructive pulmonary disease (2). An epidemiological study (2) found that more than 64 million patients suffered from heart failure of different severity globally, associated with a heavy economic burden of over 346 billion US dollars. Due to the pathological changes in cardiac structure or function (3, 4), patients with heart failure usually experience distress such as dyspnea, edema, nocturnal dyspnea, fatigue, and exercise intolerance, which could impact on patients’ daily living (4) and socialization (5), and even result in mental health problems such as depression.

Heart failure in patients is associated with a higher risk of depression (3, 6). A systematic review and meta-analysis indicated that 41.9% of heart failure patients suffered from depression, and 28.1% had moderate to severe depression (7). The prevalence of depression is also related with the severity of heart failure. For instance, the prevalence of depression was 54.7% among patients diagnosed with stage III and stage IV heart failure according to the New York Heart Association (NYHA) classification, which was higher than the figure (32%) in those with stage I and II heart failure (7). In addition, depression is a significant risk factor for increased morbidity (8) and mortality among heart failure patients (9–11). The mortality risk in heart failure patients with depression was more than 50% (11) to twice (12) higher than in heart failure patients without depression, which is not surprising given that depression could affect physiological (13, 14) and behavioral systems (1). Depression could trigger the neuro-hormonal activation *via* increasing the activity of the hypothalamic–pituitary–adrenal axis, resulting in hypercortisolemia, high blood pressure, arrhythmias, hypercoagulability and inflammatory markers-cytokines release (13, 14), all of which might negatively influence the prognosis of heart failure. Furthermore, depression could adversely affect heart failure patients’ treatment adherence (15), functional status, self-care ability (16), social support (8, 17), and quality of life (18–20).

Heart failure patients with comorbid depression were found to have a high burden on their caregivers (21) and frequent hospitalizations, resulting in 22% (22) to 30% (23) increased cost to the health care system. Although the European Society of Cardiology (ESC) and American Heart Association (AHA) guidelines for heart failure recommend early detection of depression among heart failure patients during hospitalization and follow-up (1, 4), implementation in clinical practice remains challenging. Identifying depression in heart failure patients is difficult due to their overlapping symptoms such as sleep disturbance, fatigue, and loss of appetite (8, 15, 24). Moreover, the effectiveness of antidepressants on depressed patients with heart failure remains unclear (8). Several meta-analyses on non-pharmacological therapies, such as exercise training (25), psychological interventions (26) and cognitive-behavioral therapy (27), showed positive effects on depressive symptoms (depression hereafter) among heart failure patients. Hence, providing an overall perspective on the research related to heart failure and depression is vital for understanding the current development, hotspots and frontiers in this healthcare research field. However, the information in this ‘areas’ is still lacking.

Bibliometrics is a widely used scientific method to examine research development (28), and can help researchers assess the scientific information rapidly, and provide reliable and practical knowledge development in a specific research field (28, 29). Bibliometrics perform quantitative and qualitative analyses on different characteristics of scientific publications, and then provide a comprehensive overview of the current development, hotspots, and emerging trends in the particular field by identifying the frequency of publications, citations, and commonly used keywords (30, 31). Moreover, bibliometrics could distinguish the cooperative relationships between countries, institutions, and authors, which provide valuable information on knowledge clusters to researchers with structural networks (29). Therefore, bibliometrics may empower researchers to identify the research gap and develop new ideas that can contribute new directions in the respective field (30). With the rapid development in psycho-cardiology, the number of publications related to this field has increased steadily (32). To date, several bibliometric analyses on various psycho-cardiology topics have been published. For instance, one study examined the trend of 157 publications on atrial fibrillation and depression published between 2001 and 2021 (33), while another study examined 8,073 articles published between 2004 and 2020 on coronary heart disease and depression/anxiety (34). However, no bibliometric analysis of the research on heart failure and depression has been published. To fill this gap, this study comprehensively explored the patterns of research on heart failure and depression from the perspective of bibliometric analysis.

## 2. Methods

### 2.1. Data acquisition and search strategy

**Figure 1:**
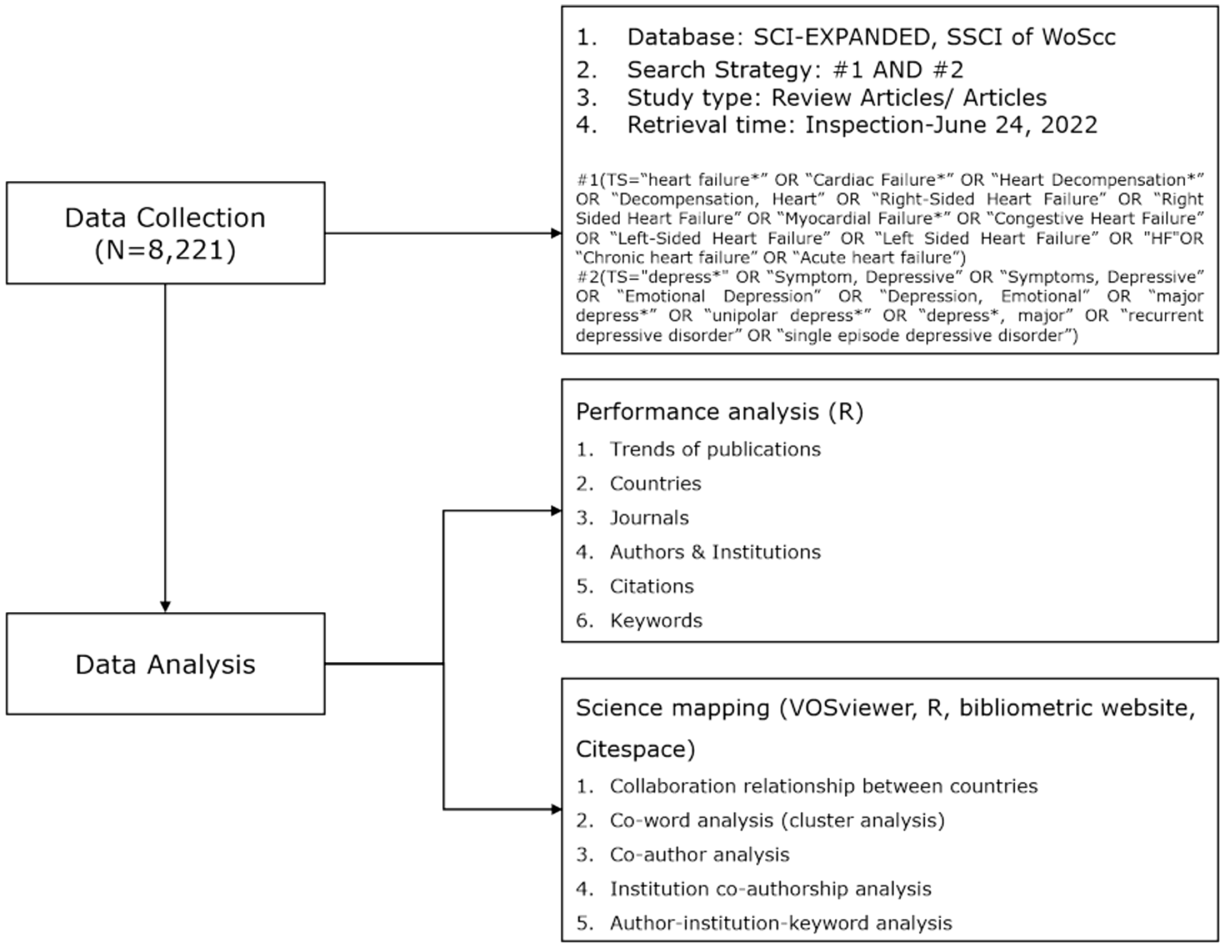
Flowchart of data collection and study design.

Web of Science (WoS) is one of the most commonly used databases for the purpose of bibliometric analysis (35–37). With about 12,000 academic journals and accessible and informative bibliometric indicators, WoS provides the most complete and dependable data for bibliometric analysis (38, 39). For this study, the Sciences Citation Index (SSCI) and Science Citation Index Expanded (SCI-expended) in the Web of Science Core Collection database (WoSCC) were searched from their inception dates to June 24, 2022. Search terms were based on the Medical Subject Headings (MeSH) terms and relevant publications on heart failure and depression, including: (TS = heart failure*” OR “Cardiac Failure*” OR “Heart Decompensation*” OR “Decompensation, Heart” OR “Right-Sided Heart Failure” OR “Right Sided Heart Failure” OR “Myocardial Failure*” OR “Congestive Heart Failure” OR “Left-Sided Heart Failure” OR “Left Sided Heart Failure” OR “HF” OR “Chronic heart failure” OR “Acute heart failure) AND (TS = “depress*” OR “Symptom, Depressive” OR “Symptoms, Depressive” OR “Emotional Depression” OR “Depression, Emotional” OR “major depress*” OR “unipolar depress*” OR “depress*, major” OR “recurrent depressive disorder” OR “single episode depressive disorder”). The types of literature included reviews and original articles, with no limitation in the language of the publication. Figure 1 shows the flow chart of data collection.

### 2.2. Data analysis

The software of R version 4.2.1 (40), VOSviewer version 1.6.18 (41), and Citespace version 6.1.R2 (42) and the bibliometric website were used for the bibliometric analysis.

The *Bibliometrix* package of R (40) was applied to perform comprehensive quantitative and science mapping analysis on bibliometric research. In this study, *Bibliometrix* was used to (1) analyze the bibliometric indicators, including the trends of publications, and related countries, journals, articles, authors & institutions, keywords, and citations; (2) estimate the frequency of international collaborative relationships between countries; (3) identify the relationships between authors, institutions and keywords by using the three-field plot. The number of annual articles on the research field of heart failure and depression between 2022 and 2030 was predicted by linear regression analysis (43, 44) using R (45), and the visualization map of collaborative relationships between countries was analyzed by a free online bibliometric website.^1^

VOSviewer program is a commonly used scientific visual mapping tool based on the distance method by bibliometric indicators (46). The node size shows the strength of indicators such as the number of publications or frequency of keywords; the link between nodes indicates the relationships between nodes such as institutions, keywords and authors, which is evaluated by the total link strength. Shorter distance between two nodes represents more substantial relationship between the nodes, while colors indicate different communities with closer associations (34, 38, 39, 47). Additionally, VOSviewer software contains the function of the overlay visualization map, which can interpret the transformation in the specific field of research, with the color range from purple (previously), green to yellow (recently) (47). VOSviewer software in this study was used to illustrate the network and overlay visualization map on co-word analysis, co-author analysis, and institution co-authorship analysis.

CiteSpace is a java application software used to utilize a dynamic visualization map, analyze the directions and features of bibliographic data (39) and provide critical analysis in the field of research development (40). CiteSpace was used to illustrate the strongest citation bursts of keywords during a specific period.

## 3. Results

### 3.1. Summary of publications

A total of 8,221 publications in the heart failure and depression research field were identified, which were published between 1983 and 2022, and consisted of 7,376 original and 845 review articles. Among these articles, most (7,964; 96.8%) were published in English, followed by articles in German (84), Spanish (48), Russian (47), French (36), Chinese (10), Japanese (7), Polish (6), Portuguese (6), Italian (3), Turkish (2), and one article each in Dutch, Korea, Hungarian, Slovenian, Icelandic and Serbian.

**Figure 2:**
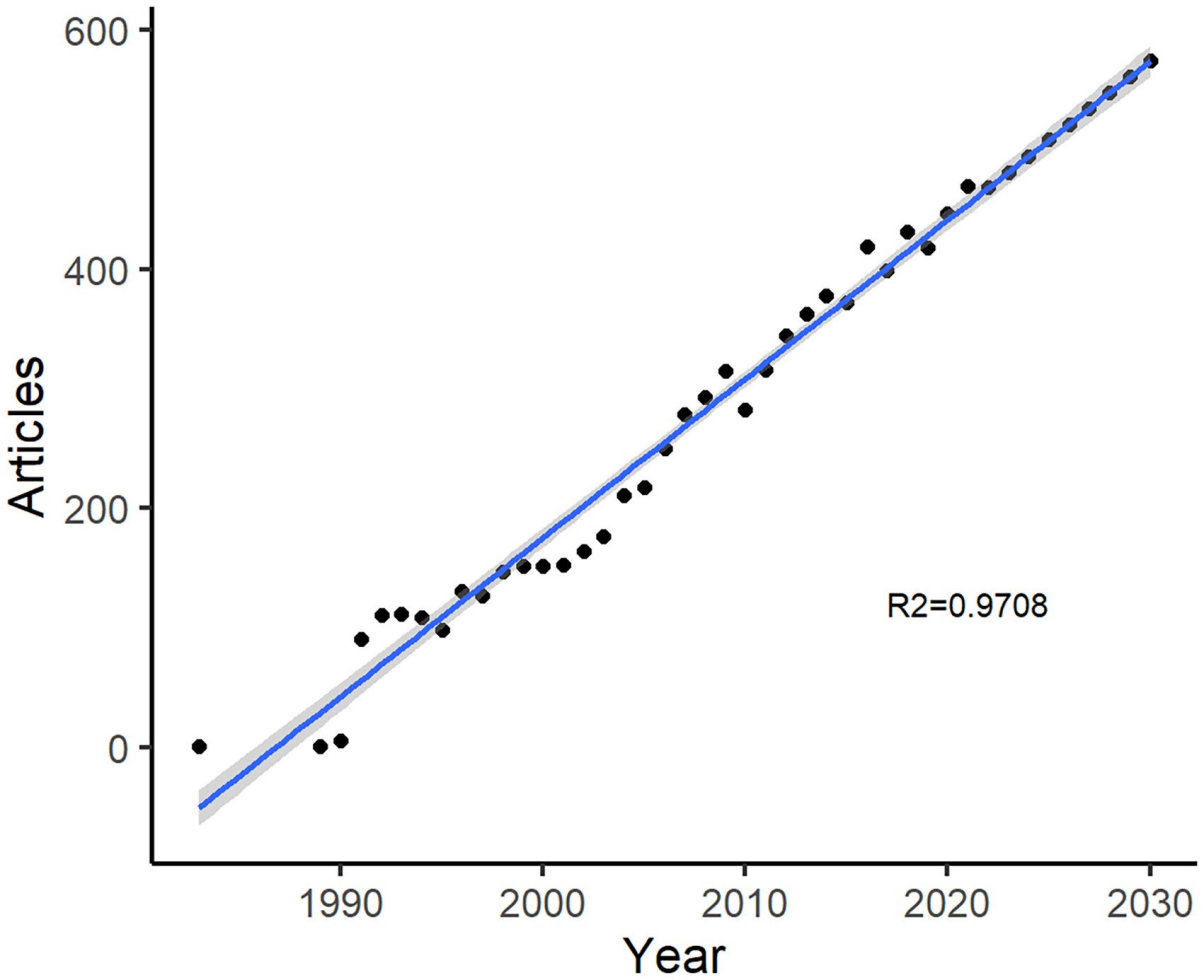
Number of annual articles on heart failure and depression by years.

Figure 2 displays the upward trend in terms of the number of articles published annually. The annual growth rate was 14.79%. The first article was published in 1983, followed by a rapid growth in publications from 1 to 91 (growth rate: 910%) between 1989 and 1991. In the following decade, articles increased steadily to 164 in 2002 (growth rate: 48%), whereas in the following two decades, the number of articles increased to 469 by 2021. Figure 2 illustrates the predicted growth trend of the article from 2022 to 2030, according to the generalized linear model used to measure the relationship between the annual numbers of articles and publication year (by excluding the period from 1984 to 1988 when there were no publications, and also 2022), with the fitness of predicted model (R^2^ = 0.9708). The predicted result revealed a stable growth trend; with the expected number to be 574 in 2030. Supplementary Table S1 shows the details of the prediction.

### 3.2. The most productive countries

**Figure 3:**
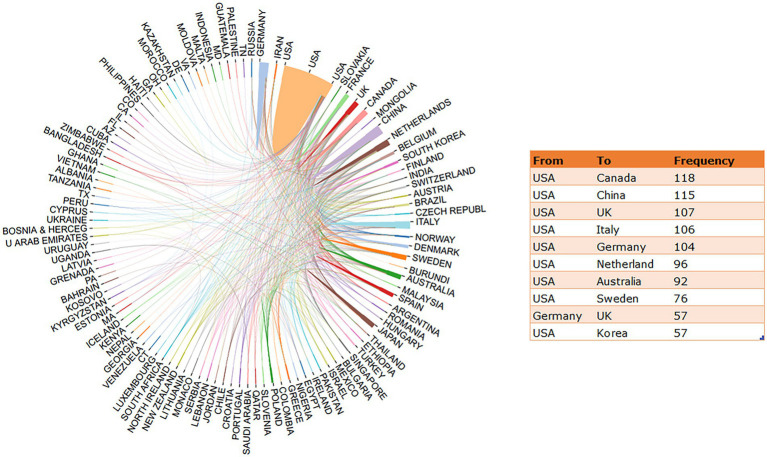
The collaborative research between countries on heart failure and depression.

In case where only corresponding authors were included, a total of 106 countries contributed to the publications on heart failure and depression. Table 1 shows the top 10 most productive countries on research related to heart failure and depression. The most productive country was the United States, which accounted for 36.65% of the total publications [*N* = 3,013; Total Citation (TC) = 149,376], followed by China (*N* = 570; 6.93%; TC = 8,093), Germany (*N* = 534; 6.50%; TC = 16,062), Italy (*N* = 384; 4.67%; TC = 14,055), and Japan (*N* = 335; 4.07%; TC = 7,677). The publications from the remaining countries contributed less than 4% of the total number of articles. Figure 3 shows the collaborative research between countries of all authors on heart failure and depression. International collaborations on the related research field is widely spread across North America, European, Asia and Oceania. The United States is the most active country cooperating with other countries, particularly with Canada (frequency = 118), followed by China (frequency = 115) and the United Kingdom (frequency = 107).

**Table 1:** Top 10 most productive countries on research related to heart failure and depression.

SCR	Country	Articles	Percent	TC	AAC	SCP	MCP	MCP_Ratio
1	United States	3,013	36.65%	149,376	49.58	2,583	430	0.14
2	China	570	6.93%	8,093	14.20	471	99	0.17
3	Germany	534	6.50%	16,062	30.08	402	132	0.25
4	Italy	384	4.67%	14,055	36.60	290	94	0.24
5	Japan	335	4.07%	7,677	22.92	306	29	0.09
6	Canada	316	3.84%	13,449	42.56	226	90	0.28
7	Netherlands	314	3.82%	14,893	47.43	195	119	0.37
8	United Kingdom (UK)	287	3.49%	15,473	53.9	191	96	0.33
9	France	195	2.37%	7,810	40.0	146	49	0.25
10	Sweden	195	2.37%	4,886	25.0	123	72	0.37

SCR: Standard Competition Ranking; TC: Total Citations; AAC: Average Article Citations; SCP: Single Country Publications; MCP: Multiple Country Publications.

### 3.3. The most productive institutions and cooperation

**Figure 4:**
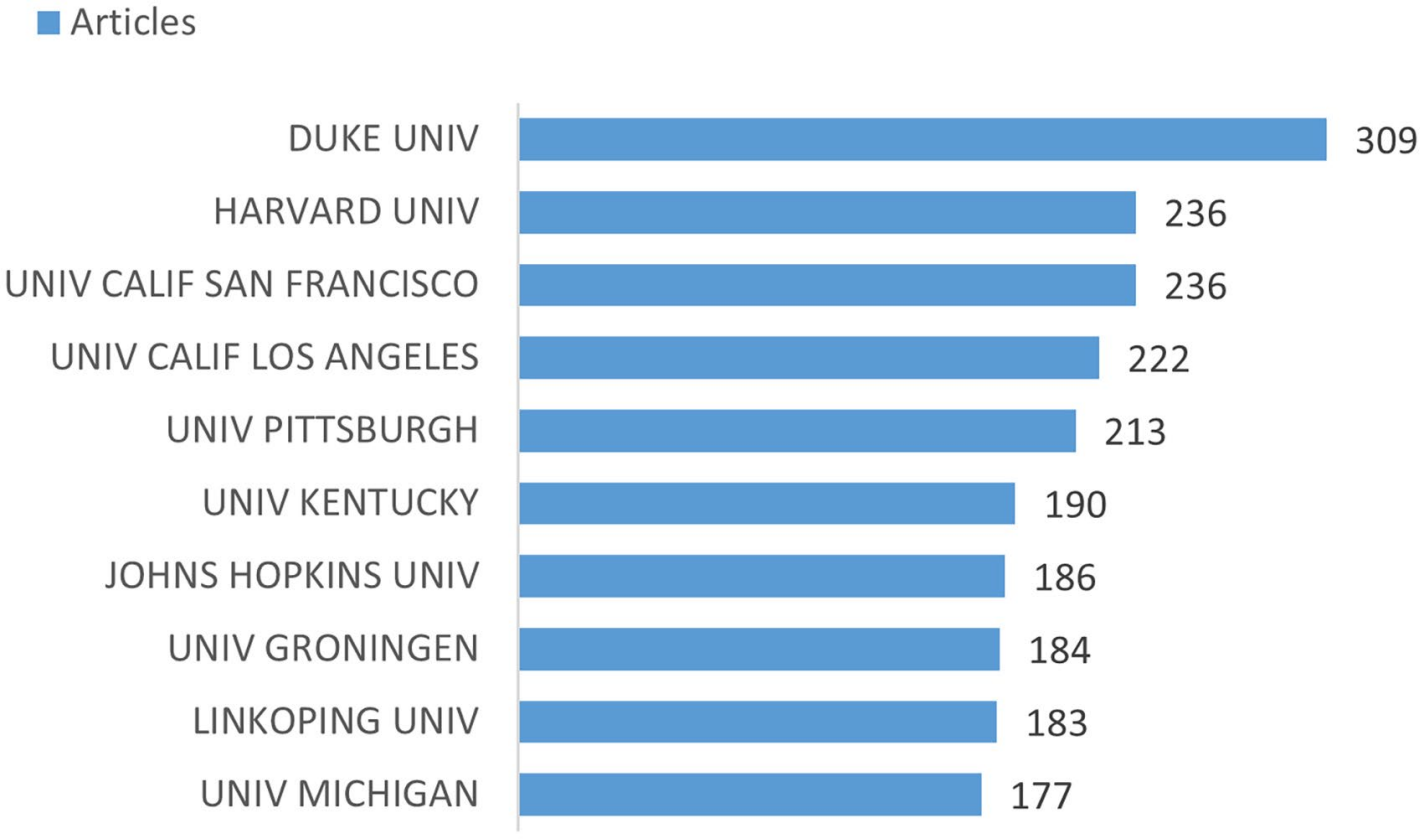
Top 10 institutions with most publications in heart failure and depression.

In total, 6,667 institutions contributed to the research on heart failure and depression. Figure 4 summarizes the top 10 institutions with the most publications, with 8 of the top 10 institutions being located in the United States, while one is located each in the Netherland and Sweden. The top 3 most productive institutions on heart failure and depression research were Duke University (*N* = 309), Harvard University (*N* = 236), and the University of California San Francisco (*N* = 236). The co-authorship analysis between different institutions evaluated the underlying collaborative relationship. As shown in Supplementary Figure S1A, with a minimum number of 60 publications in each institution, the size of the node presents the number of articles, and the color indicates different communities. Forty institutions were included covering four communities in the network visualization map, which represented stable scientific collaborations between institutions. Duke University’s research team was placed in the center of the map and closely connected with all other communities, with the largest total link strength of 174.

Institution co-authorship analysis of research on heart failure and depression could outline the transformative cooperation over time as displayed in Supplementary Figure S1B, with the color indicating the average publication year in the specific research field. The visualization marked in purple reveals the average publication year of institutions that started earlier, while the green to yellow represents the average publication year of the institutions that began more recently. Research teams in both Harvard University and University of California Los Angeles started the specific field of research in early 2010, followed by research teams in Duke University which started between 2010 and 2011. The University of Kentucky and Linkoping University have conducted research on heart failure and depression more recently than other institutions. Further, Harvard University (*N* = 177, average publication year = 2003) and University of California Los Angeles (*N* = 128, average publication year = 2009) had more publications in earlier periods.

### 3.4. The higher-impact journals

Altogether, 8,221 articles on heart failure and depression were published in 1,774 journals. Table 2 shows the top 10 journals with a total number of publications of 1,317, accounting for 16% of the total publications. Based on the categories in Journal Citation Report (JCR), the majority of the top 10 journals was located in Q2 (50%), followed by Q1 (30%) and Q3 (20%), with 90% of the journals being classified as Cardiology and Cardiovascular Nursing. Most of the top 10 journals were in the United States (7), followed by one each in Ireland, the United Kingdom, Netherlands, respectively. The number of articles published in the top 10 journals ranged from 100 to 184; Circulation (2021 IF = 39.918) was the most productive journal with 184 articles, followed by the American Journal of Physiology-Heart and Circulatory Physiology (2021 IF = 5.125) with 166 articles, and the American Journal of Cardiology (2021 IF = 3.133) with 160 articles.

**Table 2:** Top 10 journals with most publications in the field of heart failure and depression.

SCR	Sources	Articles	Country	IF	JCR
1	Circulation	184	United States	39.918	Q1
2	American Journal of Physiology-Heart and Circulatory Physiology	166	United States	5.125	Q2
3	American Journal of Cardiology	160	United States	3.133	Q3
4	Journal of the American College of cardiology	129	United States	27.203	Q1
5	Journal of Cardiovascular Nursing	126	United States	2.468	Q3
6	Journal of Cardiac Failure	122	United States	6.592	Q2
7	International Journal of Cardiology	112	Ireland	4.039	Q2
8	European Journal of Cardiovascular Nursing	109	United Kingdom	3.593	Q2
9	European Journal of Heart Failure	109	Netherlands	17.349	Q1
10	Plos One	100	United States	3.752	Q2

IF, impact factor (2021–2022); JCR-c, Journal Citation Reports category (2021).

### 3.5. The most influential authors and institutions

A total number of 35,099 authors contributed to published articles on heart failure and depression. Table 3 shows the top 10 most productive authors with the number of publications, institution, H-index, and total citations. Interestingly, 60% of the top 10 most productive authors came from the institutions involving nursing. Debra Moser was the most productive author from the University of Kentucky, with 107 articles (H-index =36, TC = 4,121), followed by Johan Denollet from Tilburg University, with 68 articles (H-index = 31, TC = 3,244), and Terry Lennie from University of Kentucky, with 67 articles (H-index = 27, TC = 1,954). Supplementary Figure S2A shows the cooperative relationships between authors, based on the minimum number of publications of 14 from each author. In the network visualization map, 52 authors were presented involving 6 communities, with each clustered around at least one productive author. The largest node was Debra Moser, with the total link strength of 250, which is connected to the communities of Tiny Jaarsma, Johan Denollet, and Christopher Lee. The tendency towards co-authorship by time overlay is shown in Supplementary Figure S2B.

**Table 3:** Top 10 most productive authors contributing to research related to heart failure and depression.

SCR	Author	Institution	NP	H-index	TC
1	MOSER DK Moser, Debra K	University of Kentucky (College of Nursing)	107	36	4,121
2	DENOLLET J Denollet, Johan	Tilburg University (Department of Medical and Clinical Psychology)	68	31	3,244
3	LENNIE TA Lennie, Terry A	University of Kentucky (College of Nursing)	67	27	1,954
4	JAARSMA T Jaarsma Tiny	Linköping University (College of Nursing)	66	29	2,700
5	PEDERSEN SS	University of Southern Denmark (Department of Psychology)	61	27	1,737
6	DHALLA NS	University of Manitoba (College of Medicine)	54	25	2,584
7	STROMBERG A	Linköping University (College of Nursing)	47	21	1,544
8	CHUNG ML	University of Kentucky (College of Nursing)	45	22	1,334
9	RIEGEL B	University of Pennsylvania (School of Nursing)	45	28	3,314
10	BAEKEN C	Eindhoven University of Technology (Department of Electrical Engineering)	44	21	2,506

NP: Number of publications.

### 3.6. Research hotspots

#### 3.6.1. Most cited articles

Analysis of the most cited articles displays crucial information about a specific research field, and the total citation (TC) numbers reflects articles’ importance (34). Supplementary Table S2 lists the top 10 most cited articles in the research field of heart failure and depression with more than 1,000 citations, which were published between 1994 and 2014. The top 10 most cited articles mainly focused on chronic diseases and their comorbidities such as cardiovascular diseases, pulmonary diseases and depression.

Pedersen and Saltin (49) published the most cited article (TC = 1,205) that summarized the evidence on different types and strengths of exercise as a treatment to improve chronic diseases, such as metabolic syndrome disease, cardiac disease and depression. The second most cited article was published by Naylor et al. (50) that is a randomized clinical trial study (TC = 1,185) that found a positive effect of nurse-centered discharge care plan on 363 hospitalized patients, including chronic heart failure and depression patients, to reduce the readmission rate. The third most cited article (TC = 1,132) was a longitudinal cohort study conducted by Guccione et al. (48), which showed that 7 aspects of functional limitations were significantly related to stroke, and 5 aspects of functional limitations were associated with depression and hip fracture.

#### 3.6.2. Most frequent keywords

**Figure 5:**
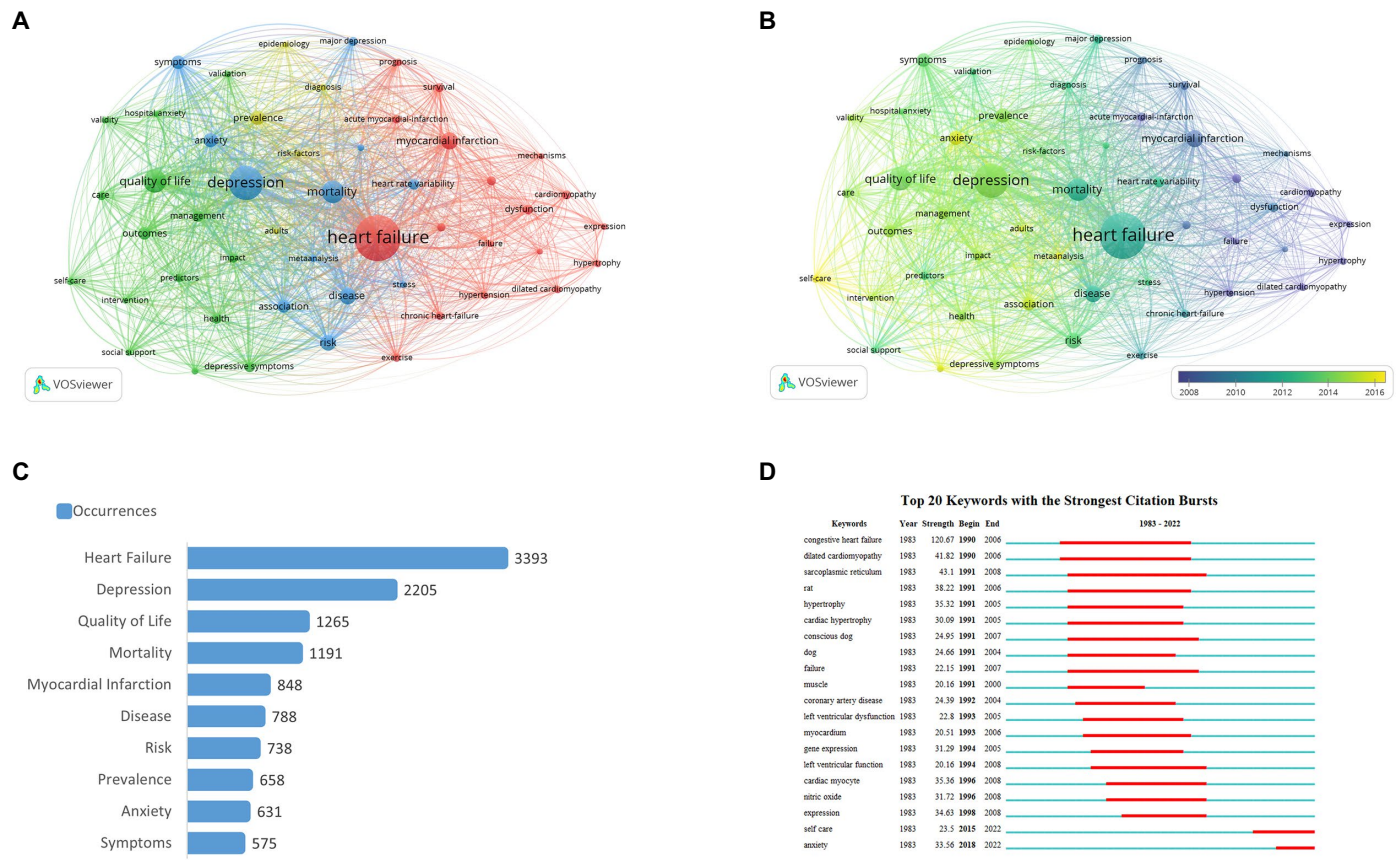
Notes: The size of each node represents the frequency of a keyword, while the link between nodes indicates the relationship between nodes, with the distance between them indicating the strength of the connection. The color indicates the co-occurrence of keywords (Figure 5A), while the color change indicates the time change of keywords (Figure 5B). ‘Begin’ and ‘End’ refer to the start and end of the keyword emergence, respectively. Strength is a measure of the intensity of the cited change. Red or blue bars represent time intervals. Red bars indicate bursts of citations (Figure 5D). **(A)** Network visualization map of keywords co-occurrences on heart failure and depression. **(B)** Network visualization map of the current tendency towards heart failure and depression based on keywords analysis. **(C)** Top 10 most frequent keywords. **(D)** Top 20 keywords with the strongest citations bursts.

Keyword analysis could show the hotspots and trends in the specific field of research. Among the 22,261 keywords extracted from the 8,221 articles, the top 10 most frequently used keywords are listed in Figure 5C, all of which have appeared more than 500 times, including “heart failure” (*N* = 3,393), “depression” (*N* = 2,205), “quality of life” (*N* = 1,265), “mortality” (*N* = 1,191), “myocardial infarction” (*N* = 848), “disease” (*N* = 788), “risk” (*N* = 738), “prevalence” (*N* = 658), “anxiety” (*N* = 631), “symptoms” (*N* = 575).

Using a minimum number of 188 in keyword occurrences, there were 50 most frequently occurring keywords which showed stable associations consisting of 4 clusters in a network visualization map as illustrated in Figure 5A. The larger the size of the node the more occurrences of the keywords, while the link between two nodes shows their connection, with the distance between them indicating the strength of the connection. The keywords co-occurrence analysis reflected the essential topics on heart failure and depression. Cluster 1, in red color, focused on heart failure and cardiovascular diseases such as “heart failure,” “myocardial infarction,” and “hypertension.” Cluster 2, in green color, concentrated on treatments of heart failure and depression, with frequently occurring keywords such as “quality of life,” “management,” and “outcomes.” Cluster 3, in blue color, was located in the center of the network and focused on outcomes of heart failure and depression, with the top keywords of “depression,” “mortality,” and “anxiety.” Cluster 4, in yellow color, concentrated on epidemiology related to heart failure and depression, with the main keywords of “epidemiology,” “prevalence,” and “risk factors.” However, the hotspots of related research fields have changed over time as shown in Figure 5B which illustrates the current tendency towards heart failure and depression based on keywords analysis by time overlay. The purple color presents the keywords widely used before 2008, whereas the yellow color presents those widely used from 2015 to present. The keyword “myocardial infarction” was predominantly used from early 2008 to 2010, while “heart failure” has been widely used since late 2010; the keywords “depression,” “quality of life,” and “management” have been used more recently since 2014, which indicate the current and future trend of research in this field.

Supplementary Figure S3 displays the relationships between authors, institutions and keywords occurrence in the research field of heart failure and depression. The research team of Debra Moser at University of Kentucky, cooperated with the research team of the University of California San Francisco and the University of California Los Angeles frequently, with heart failure and depression as the mutual keywords.

#### 3.6.3. Keywords with citation burst

Figure 5D shows the top 20 keywords with the strongest citation bursts. The keywords of “congestive heart failure” (1990–2006), “dilated cardiomyopathy” (1990–2006), “sarcoplasmic reticulum” (1991–2008), “failure” (1991–2007) and “conscious dog” (1991–2007) presented as hotspots for a long period in the past; whereas “self-care” (2015–2022) and “anxiety” (2018–2022) have been used more recently, which indicate the future hotspots in the research field of heart failure and depression.

## 4. Discussion

To the best of our knowledge, this is the first bibliometric analysis on heart failure and depression to explore the development of research related to heart failure and depression over the past 33 years. The number of articles published on heart failure and depression showed an upward trend over the past decades. Previous bibliometric analysis on coronary heart disease and depression (34) and psycho-cardiological disease (32) also found growing trends over time, both of which are consistent with our findings. The increasing interest in the relevant research field is possibly associated with the growing importance of mental health and social burden among patients with chronic diseases (51). In addition, psycho-cardiology has gained growing attention (72), probably reflecting the overlapping pathophysiological mechanisms between psychosocial factors and cardiovascular diseases (14).

The bibliometric analysis revealed that the most productive countries (80%) are from North America and Europe such as the United States, Canada, Germany, and Italy, which is consistent with previous findings of bibliometric studies on depression and coronary heart disease (34), atrial fibrillation (33), and psycho-cardiological disease (32). Most of the productive countries are from high-income countries with more research fundings in academic research compared to other areas; for example, the United States, Germany, France and United Kingdom were among the top contributing countries in global research and development during the past two decades (52). Moreover, the prevalence of cardiovascular patients with depression appeared to be higher in high-income countries compared to middle to lower-income countries (53), which may increase academic attention in this area. Finally, the growth of publications among developed countries may be related to the location of key authoritative global organizations; for example, the American Heart Association and the European Society of Cardiology, both of which invested billions of funding in research to promote evidence-based scientific guidelines to reduce the harm and burden of cardiovascular diseases (54, 55).

The United States was the leading country in this research field of heart failure and depression, with the largest number of publications and citations, which is potentially associated with the level of support for academic research. The US’s expenditure on medical and health research development increased from approximately USD 6.54 billion in 1983 to more than USD 60.15 billion in 2020 (56). The rapid growth trend in the past decades could partly explain the highest proportion of total publications in the United States. Other countries, such as China, also contributed substantially to the heart failure and depression research, possibly due to the increased research expenditures. Over the period 2000 to 2019, China contributed 29% of the growth in global research and development, with more than 10% annual growth (52). However, the total citations and annual citations of publications in China were still lower than the several European countries (e.g., Germany, Italy), which suggests that research quality in China may take more time to improve as the quantity of academic research increases. Additionally, there were less international collaborations in China with other countries and international institutions. In contrast, the international collaborations in heart failure and depression research is strongly centered in the United States; for instance, Duke University, located in the United States, played a critical leading role in cooperation between numerous institutions. Therefore, international collaboration in research between countries and institutions outside the United States should be enhanced.

Identifying current publishing trends in a specific research field could identify the features of high-impact journals in the field (39). In this study, 80% of the top 10 most productive journals are located in the United States. Even though China and Japan were two of the major countries in heart failure and depression research, no east Asian publishers are among the top 10 countries. Therefore, it is necessary to improve the impact of academic journals in Asia. The Journal impact factor is an important indicator of journal quality based on citation frequency over time (57). Journal Citation Report (JCR) is also widely used to evaluate the global impact of journals based on the rank of journal impact factors divided into four quartiles, ranging from the most influential (Q1) to the least influential (Q4) journals (58). Circulation was the most influential journal in heart failure and depression research, with the highest impact factor and the most number of relevant publications in this area. In contrast, 50% of the top 10 most productive journals had an impact factor lower than 5, while 80% are in Q2 (50%) and Q3 (20%).

In authorship analysis, research works of leading authors could reflect the influential knowledge structure in a particular field (33). The United States was predominant in terms of productive authors; for instance, 6 out of the top 10 productive authors worked in the United States. In addition, the research teams of Debra Moser, Tiny Jaarsma, Susanne Pedersen, and Christopher Lee, who work in nursing institutions in the United States, played the leading roles in this research area as indicated by the collaborative authorship communities on the network visualization map. The analysis of cooperative relationships between productive research teams could provide useful information to potential collaborators (59). The field of psycho-cardiological diseases involves interdisciplinary treatment and care provided by a multidisciplinary team including physicians, nurses, psychologists, psychiatrists, and physiotherapist (32). Thus, international collaboration between researchers from different disciplines and countries should be strengthened to promote research development.

Research hotspots may reflect academic topics in a specific research field over a certain period, which is a critical indicator in bibliometric analysis (60). The number of publication citations could reflect academic influence. In this study, most cited articles focused on chronic diseases such as cardiovascular diseases and depression. The articles published by Pedersen and Saltin (49) and by Booth, Roberts (61) that examined exercise effects on chronic disease were among the top 10 most cited papers, reflecting the academic research trends. Pedersen and Saltin (49) concluded that exercise as a treatment could partly improve chronic diseases, while Booth, Roberts (61) summarized the evidence that physical inactivity was a significant cause of chronic diseases through alteration in the arterial and endothelial system function. Thus, the most cited articles could add to the knowledge development in the treatment of heart failure and depression. For instance, a meta-analysis conducted by Tu, Zeng (25) of 19 studies with 3,447 patients showed that depression could improve following exercise training in heart failure patients, possibly through improvements in neuro-hormonal or inflammatory systems (62). Additionally, heart failure management guidelines by the AHA (1) and ECS (4) recommended regular exercise as a routine intervention to improve functional status and quality of life among heart failure patients.

Keywords co-occurrence analysis is also a key indicator in bibliometric analysis to provide the dominant and hot topics in a specific research field (63). Apart from “heart failure” and “depression,” other widely used keywords mainly focused on the comorbidities and outcomes of heart failure and depression. In the analysis of co-occurrences of keywords clusters, each cluster had a main theme. Cluster 1, in red color, tended to focus on the risk factors of heart failure in terms of myocardial infarction and hypertension, which is consistent with previous findings that diabetes (64), myocardial infarction (65), hypertension (66), cardiomyopathy (67), depression (8) were major potential causes of heart failure. Cluster 2, in green color, mainly reflected the management and outcomes of heart failure and depression, such as “quality of life,” “self-care,” and “intervention.” Previous research found that heart failure patients with depression compared with those without depression often had poorer self-care, higher rehospitalization, lower quality of life and higher mortality (3), which might focus more attention on developing effective treatments for heart failure patients with depression.

The analysis of the keywords with the highest citation bursts in a specific research area during a period could provide an overview regarding the research development in recent periods and speculate the future trend of research direction (34). “Self-care” has been a burst keyword since 2015 as it is an important prognostic factor in heart failure patients (68). Self-care is important both in terms of treatment adherence and health maintenance in heart failure (69) often facilitated by specific education and support programs. However heart failure patients with depression commonly exhibited poor self-care behaviors (73) possibly due to a range of barriers. Previous studies found that providing knowledge through face-to-face education alone was insufficient to enhance self-care among heart failure patients with depression (70) indicating that further innovative approaches to improve self-care is needed for such patients. In addition “anxiety” has been the recent strongest burst keyword since 2018 which may be potentially associated with an article published by Celano Villegas (15). The paper reported that anxiety was linked to the development and progression of heart failure due to increasing unhealthy behaviors inflammatory responses and endothelial dysfunction. Further anxiety is negatively associated with poor self-care among heart failure patients (68). Despite the adverse effects of anxiety the management guidelines for heart failure only recommended routine depression screening for heart failure patients (1). Additionally it is challenging to make accurate diagnoses of anxiety and depression among heart failure patients due to their overlapping symptoms (15, 71). Consequently developing early detection measures and effective treatments for heart failure patients with depression or anxiety to improve their self-care is potentially an important future research hotspot

There are several limitations to this study. First, following relevant guidelines of bibliometric analysis (30) and previous studies (32–34), literature search was performed based on the WoSCC database. Although the WoSCC is one of the most influential and widely used databases to obtain academic data in bibliometric analysis (34, 39), the possibility of missing studies could not be excluded. Second, the vast majority of publications were published in English which may have led to selection bias, therefore, publications in other languages should be analyzed in the future.

In summary, the study found that the number of publications related to the research of heart failure and depression has rapidly increased over the past three decades, reflecting the growing global interest in this research field. The United States dominated the related research area, with the highest number of publications and citations. Debra Moser and Duke University were the most productive author and institution respectively, while Circulation was the most influential journal. The collaboration between countries, institutions, and researchers were mostly concentrated in the United States. The keywords of “heart failure,” “depression” and “quality of life” were research hotspots, while “self-care” and “anxiety” have attracted increasing attention recently, which may represent hotspots for future research. This bibliometric analysis on research related to heart failure and depression provides a comprehensive perspective of the current status and future trends for research in this field.

## Data availability statement

The original contributions presented in the study are included in the article/Supplementary material. Further inquiries can be directed to the corresponding authors. Requests to access the datasets should be directed to xyutly@gmail.com.

## Author contributions

ML, PC, X-MX, and Y-TX: study design. ML, PC, GL, Y-FL, TS, and GU: data collection, analysis and interpretation. ML, PC, and Y-TX: drafting of the manuscript. CN: critical revision of the manuscript. All authors contributed to the article and approved the submitted version.

## Funding

The study was supported by the National Science and Technology Major Project for investigational new drug (2018ZX09201-014), the Beijing Municipal Science & Technology Commission (No. Z181100001518005), and the University of Macau (MYRG2019-00066-FHS; MYRG2022-00187-FHS).

## Conflict of interest

The authors declare that the research was conducted in the absence of any commercial or financial relationships that could be construed as a potential conflict of interest.

## Publisher’s note

All claims expressed in this article are solely those of the authors and do not necessarily represent those of their affiliated organizations, or those of the publisher, the editors and the reviewers. Any product that may be evaluated in this article, or claim that may be made by its manufacturer, is not guaranteed or endorsed by the publisher.

## Supplementary material

The Supplementary material for this article can be found online at: https://www.frontiersin.org/articles/10.3389/fpsyt.2023.1086638/full#supplementary-material

## Footnotes

^1^ https://bibliometric.com/
