# Supplementary material for: Heart failure and depression: A perspective from bibliometric analysis
Source: Front Psychiatry. 2023 Mar 2;14:1086638. doi: 10.3389/fpsyt.2023.1086638 (PMC10017737; doi:10.3389/fpsyt.2023.1086638)
Supplement: Supplementary file 1 [file Data_Sheet_1.docx]

**Supplementary Materials**

Figure S1a. Network visualization map of institution co-authorship analysis on heart failure and depression

Figure S1b. Network visualization map for the current tendency towards heart failure and depression based on institution co-authorship analysis

Figure S2a. Network visualization map of co-authorship analysis on heart failure and depression

Figure S2b. Network visualization map for the current tendency towards heart failure and depression based on institution co-authorship analysis

Figure S3. Three-Fields Plot of the keyword analysis (Author-Institution-Keyword)

Table S1. Numbers of annual publications on heart failure and depression

| **Year** | **Articles** | **Percent** | **TC/Y** |
| --- | --- | --- | --- |
| 1983 | 1 | 0.01% | 3.15 |
| 1989 | 1 | 0.01% | 6.70 |
| 1990 | 6 | 0.07% | 0.69 |
| 1991 | 91 | 1.11% | 1.44 |
| 1992 | 111 | 1.35% | 1.62 |
| 1993 | 112 | 1.36% | 1.59 |
| 1994 | 109 | 1.33% | 2.46 |
| 1995 | 98 | 1.19% | 1.87 |
| 1996 | 131 | 1.59% | 1.65 |
| 1997 | 127 | 1.54% | 2.22 |
| 1998 | 147 | 1.79% | 2.54 |
| 1999 | 152 | 1.85% | 2.93 |
| 2000 | 152 | 1.85% | 3.67 |
| 2001 | 153 | 1.86% | 3.25 |
| 2002 | 164 | 1.99% | 3.87 |
| 2003 | 177 | 2.15% | 4.25 |
| 2004 | 211 | 2.57% | 3.96 |
| 2005 | 218 | 2.65% | 3.37 |
| 2006 | 250 | 3.04% | 3.77 |
| 2007 | 279 | 3.39% | 3.84 |
| 2008 | 293 | 3.56% | 3.52 |
| 2009 | 315 | 3.83% | 4.42 |
| 2010 | 282 | 3.43% | 3.31 |
| 2011 | 316 | 3.84% | 3.25 |
| 2012 | 344 | 4.18% | 4.23 |
| 2013 | 362 | 4.40% | 3.89 |
| 2014 | 378 | 4.60% | 4.08 |
| 2015 | 372 | 4.52% | 4.09 |
| 2016 | 419 | 5.10% | 4.38 |
| 2017 | 399 | 4.85% | 4.28 |
| 2018 | 431 | 5.24% | 4.34 |
| 2019 | 418 | 5.08% | 3.65 |
| 2020 | 446 | 5.43% | 4.30 |
| 2021 | 469 | 5.70% | 2.40 |
| 2022 | 217 (*468) | 2.64% | - |
| 2023 | *481 | - | - |
| 2024 | *494 | - | - |
| 2025 | *508 | - | - |
| 2026 | *521 | - | - |
| 2027 | *534 | - | - |
| 2028 | *547 | - | - |
| 2029 | *561 | - | - |
| 2030 | *574 | - | - |

Notes: TC/Y: Average per Year Total Citations; *The number of predicted annual publications

Table S2. Top 10 most cited articles on heart failure and depression

| **SCR** | **Author & Year** | **Title** | **Journal** | **TC** | **TC/Y** |
| --- | --- | --- | --- | --- | --- |
| 1 | PEDERSEN BK, 2006 | Evidence for prescribing exercise as therapy in chronic disease | Scandinavian Journal of Medicine & Science in Sports | 1,205 | 70.88 |
| 2 | NAYLOR MD, 1999 | Comprehensive discharge planning and home follow-up of hospitalized elders: a randomized clinical trial | JAMA | 1,185 | 49.38 |
| 3 | GUCCIONE AA, 1994 | The effects of specific medical conditions on the functional limitations of elders in the Framingham Study | American Public Health Association | 1,132 | 39.03 |
| 4 | EAGLE KA, 2004 | A validated prediction model for all forms of acute coronary syndrome: estimating the risk of 6-month postdischarge death in an international registry | JAMA | 1,095 | 57.63 |
| 5 | LEFAUCHEUR JP, 2014 | Evidence-based guidelines on the therapeutic use of repetitive transcranial magnetic stimulation (rTMS) | Clinical Neurophysiology | 1,080 | 120.00 |
| 6 | BOOTH FW, 2012 | Lack of Exercise Is a Major Cause of Chronic Diseases | Comprehensive Physiology | 1,067 | 97.00 |
| 7 | DHALLA NS, 2000 | Role of oxidative stress in cardiovascular diseases | Journal of Hypertension | 1,040 | 45.22 |
| 8 | GREEN CP, 2000 | Development and evaluation of the Kansas City Cardiomyopathy Questionnaire: a new health status measure for heart failure | Journal of the American College of Cardiology | 1,012 | 44.00 |
| 9 | HO PM, 2009 | Medication adherence: its importance in cardiovascular outcomes | Circulation | 1,002 | 71.57 |
| 10 | BARNES PJ, 2009 | Systemic manifestations and comorbidities of COPD | European Respiratory journal | 1,000 | 71.43 |

Notes: TC/Y: Average per Year Total Citations


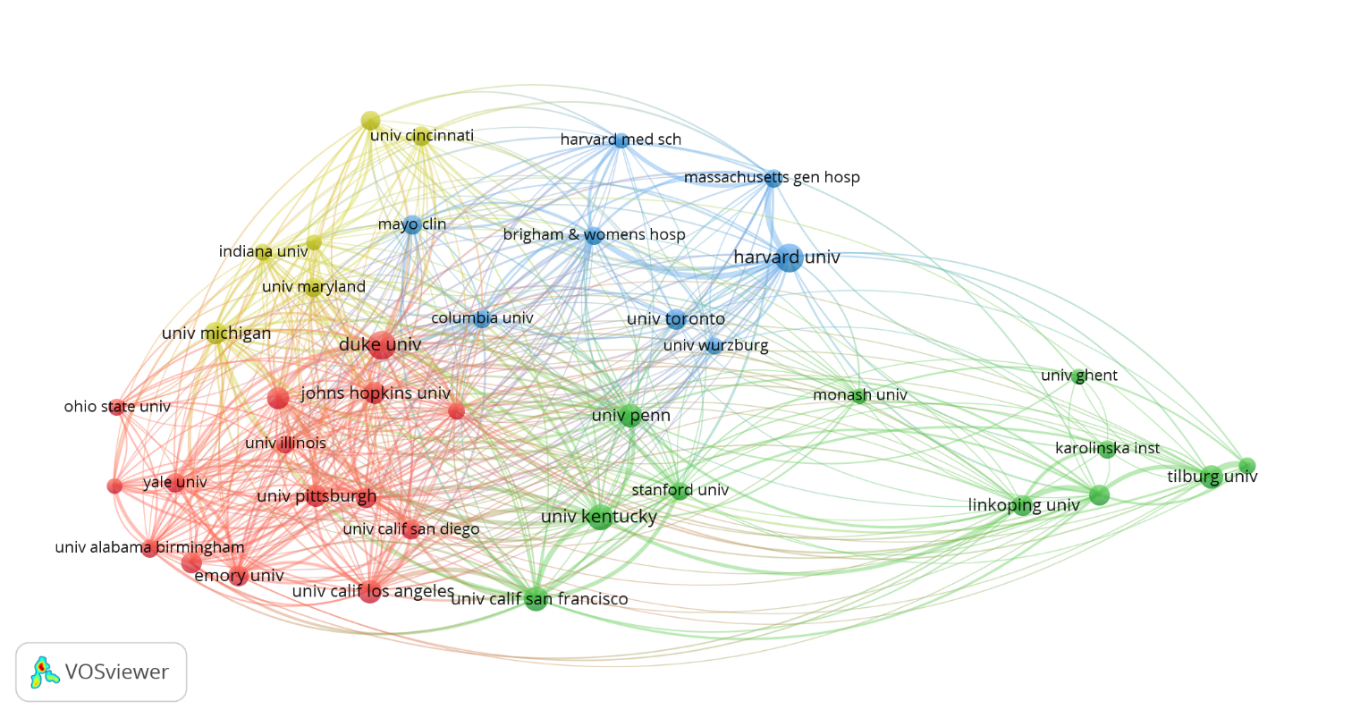


Figure S1A. Network visualization map of institution co-authorship analysis on heart failure and depression


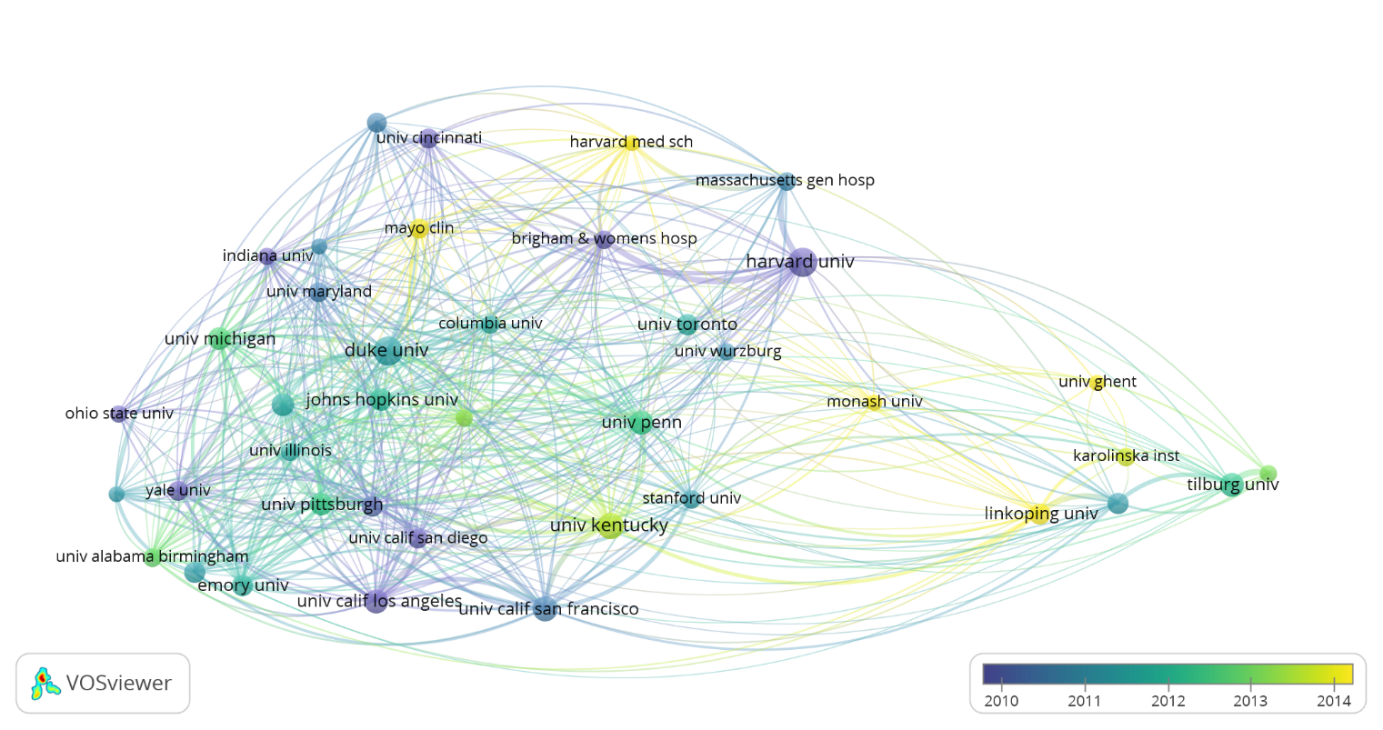


Figure S1B. Network visualization map for the current tendency towards heart failure and depression based on institution co-authorship analysis

Notes: The size of each node represents the number of articles, with a minimum number of 60 publications in each institution; the link between nodes indicates the relationship between nodes, with the distance between them indicating the strength of the connection. The color indicates different communities (Figure S1A), while the color change indicates the time change of institution’s publications (Figure S1B).


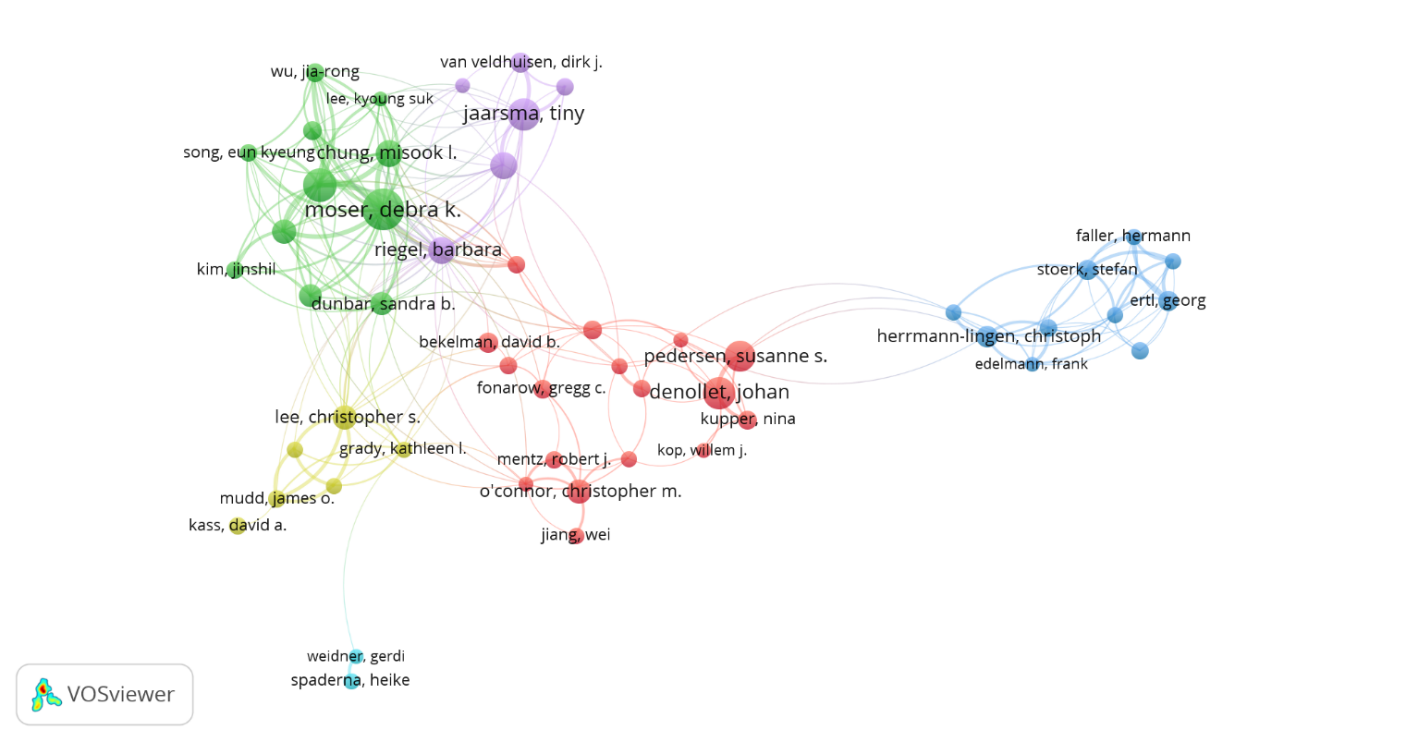


Figure S2A. Network visualization map of co-authorship analysis on heart failure and depression


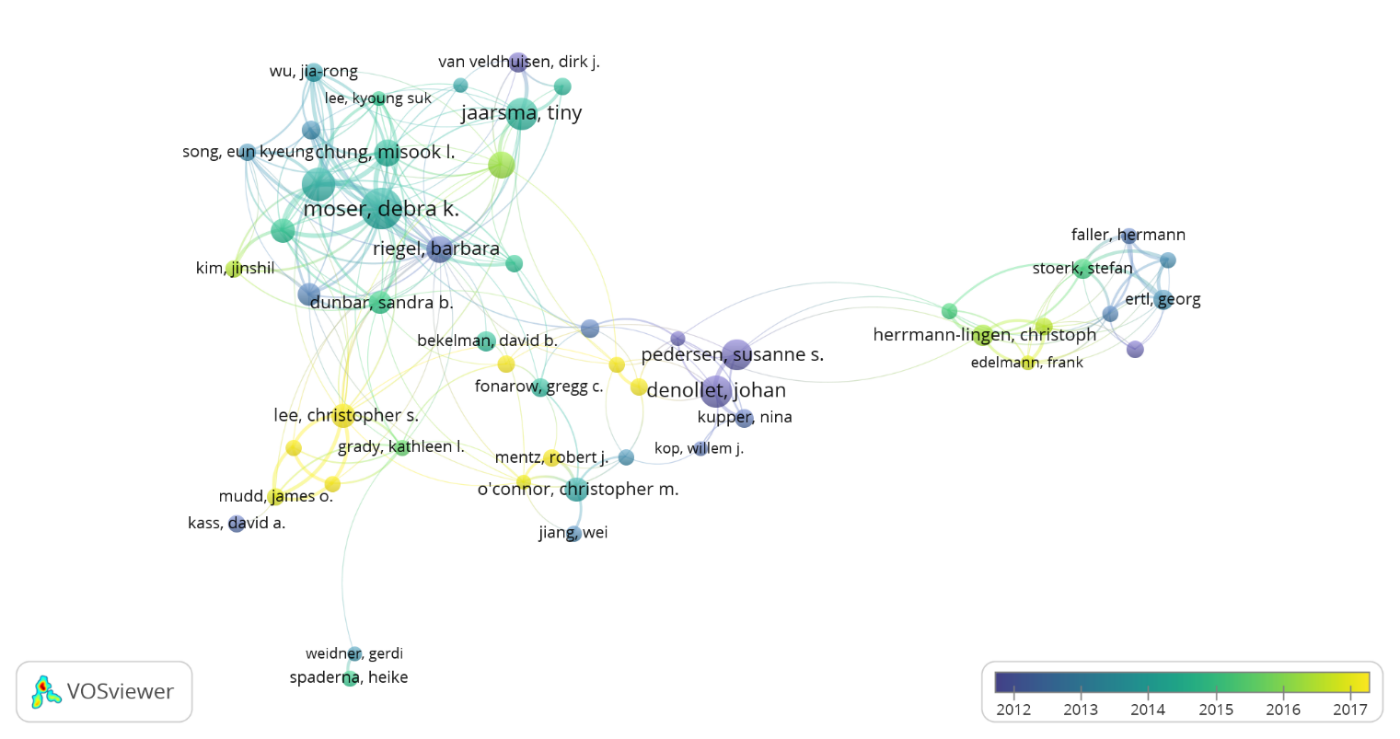


Figure S2B. Network visualization map for the current tendency towards heart failure and depression based on co-authorship analysis

Notes: The size of each node represents the strength of the respective institution co-authorship; the link between nodes indicates the relationship between nodes, with the distance between them indicating the strength of the connection. The color indicates the co-occurrence of institution (Figure S2A), while the color change indicates the time change of institution co-authorship (Figure S2B).


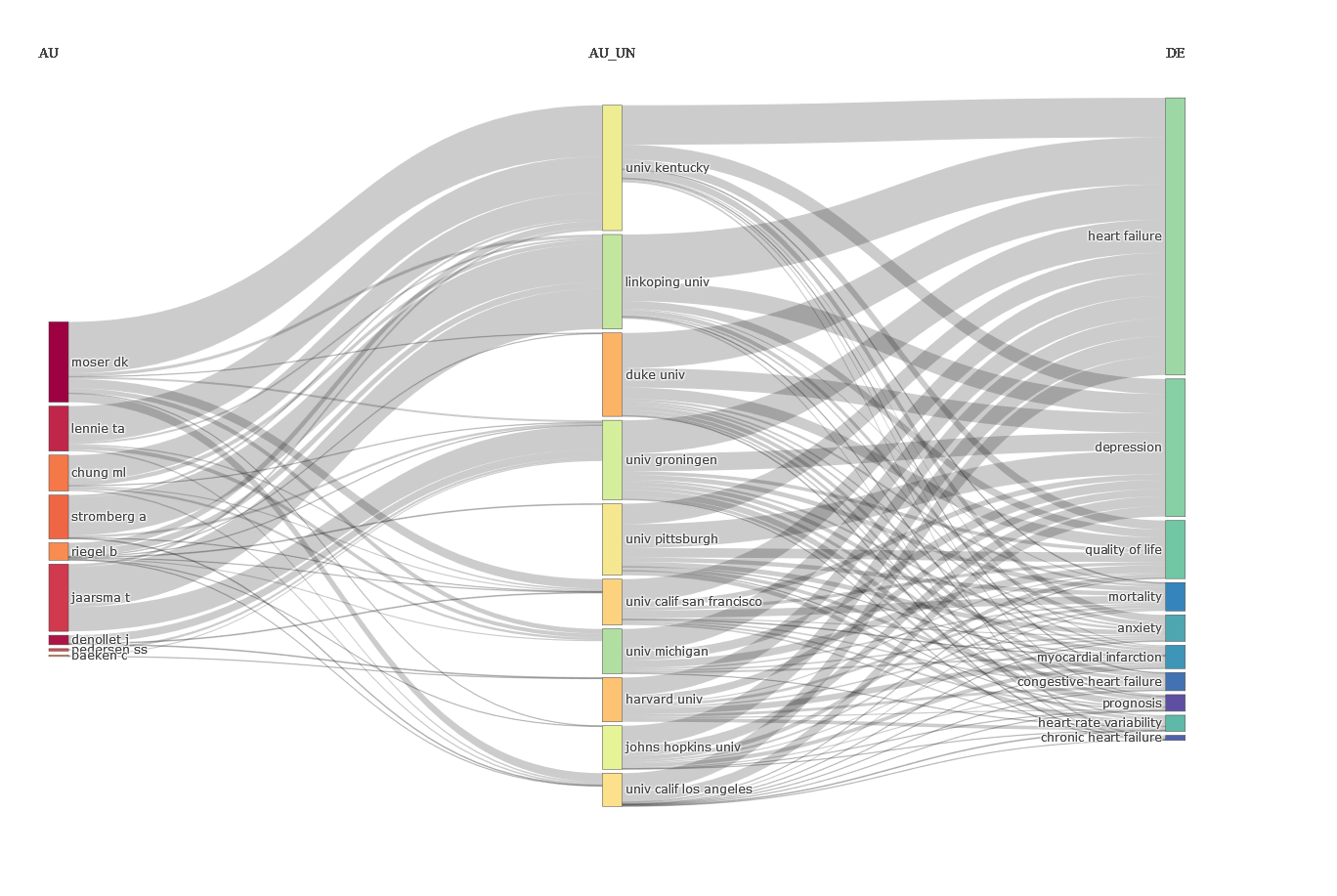


Figure S3. Three-Fields Plot of the keyword analysis (Author-Institution-Keyword)

Notes: Three-field plot of the keywords analysis: (left field: authors; middle field: affiliations; right field: keywords)
